# Isolation, Purification, and Characterization of a Laccase-Degrading Aflatoxin B1 from *Bacillus amyloliquefaciens* B10

**DOI:** 10.3390/toxins14040250

**Published:** 2022-03-31

**Authors:** Dongwei Xiong, Jun Wen, Gen Lu, Tianxi Li, Miao Long

**Affiliations:** Key Laboratory of Livestock Infectious Diseases, Ministry of Education, College of Animal Science & Veterinary Medicine, Shenyang Agricultural University, Shenyang 110866, China; 2019220557@stu.syau.edu.cn (D.X.); 2020240593@stu.syau.edu.cn (J.W.); 2019220539@stu.syau.edu.cn (G.L.); 2020240619@stu.syau.edu.cn (T.L.)

**Keywords:** aflatoxin, *Bacillus amyloliquefaciens*, laccase, degradation, molecular docking, mutagenesis

## Abstract

Aflatoxins, widely found in feed and foodstuffs, are potentially harmful to human and animal health because of their high toxicity. In this study, a strain of *Bacillus amyloliquefaciens* B10 with a strong ability to degrade aflatoxin B1 (AFB1) was screened; it could degrade 2.5 μg/mL of AFB1 within 96 h. The active substances of *Bacillus amyloliquefaciens* B10 for the degradation of AFB1 mainly existed in the culture supernatant. A new laccase with AFB1-degrading activity was separated by ammonium sulfate precipitation, diethylaminoethyl (DEAE) and gel filtration chromatography. The results of molecular docking showed that B10 laccase and aflatoxin had a high docking score. The coding sequence of the laccase was successfully amplified from cDNA by PCR and cloned into *E. coli*. The purified laccase could degrade 79.3% of AFB1 within 36 h. The optimum temperature for AFB1 degradation was 40 °C, and the optimum pH was 6.0–8.0. Notably, Mg^2+^ and dimethyl sulfoxide (DMSO) could enhance the AFB1-degrading activity of B10 laccase. Mutation of the three key metal combined sites of B10 laccase resulted in the loss of AFB1-degrading activity, indicating that these three metal combined sites of B10 laccase play an essential role in the catalytic degradation of AFB1.

## 1. Introduction

Aflatoxins are difuranocoumarin derivatives produced mainly by strains of *Aspergillus flavus* and *Aspergillus parasiticus*. They are produced during the growth and storage of crops and are chemically and thermally stable. Aflatoxin is highly hepatotoxic, nephrotoxic, acutely toxic, and immunotoxic, and belongs to a class of teratogenic, carcinogenic, and mutagenic compounds. In decreasing order of toxicity, the various metabolites are aflatoxin B1 (AFB1), AFM1, AFG1, AFB2, AFM2, and AFG2 [1,2,3,4]. Aflatoxins pose a significant risk to human health through the food chain [5].

It is essential to avoid AFB1 contamination and develop safe and effective detoxification methods to improve food safety. Traditional methods of AFB1 degradation include physical, chemical, and microbiological techniques [6]. Physical methods possess the disadvantage of being time consuming and less efficient in removing aflatoxin, while chemical methods lead to the loss of nutrients in food or feed [7]. In previous research focused on microbial degradation, AFB1 was degraded into nontoxic or less toxic metabolites by microorganisms or enzymes. The furan and lactone rings are the two key sites influencing the toxicity of AFB1 [8]. The existing studies on the microbial degradation of AFB1 also revolve around these two key sites. Some microorganisms are not probiotics, and their safety should be evaluated. Meanwhile, some microorganisms may inadvertently disrupt the nutritional properties of the product or even introduce other toxic substances that are harmful to the organism [9]. Enzymes are a promising choice, being ecofriendly and endowed with high substrate specificity and catalytic efficiency [10].

Enzymes identified to degrade aflatoxins include laccase, oxidoreductase, peroxidase, and manganese peroxidase [11]. Laccase is widely found in bacteria, fungi, insects, and higher plants [12]. Previous studies indicated that laccase can catalyse the oxidation of various compounds such as phenol, aniline, aromatic amines, ascorbic acid, and certain inorganic compounds, and can be coupled to the four-electron reduction of dioxygen to water [6,13,14,15]. The fungus laccase with high redox potential, isolated from the white-rot fungus *Cerrena unicolor* 6884, could efficiently degrade AFB1 to AFQ1 [16]. The CotA protein, as the best-known bacterial laccase, is predominantly located in the outer endospore layer of *Bacillus subtilis* and other *Bacillus* species and has a larger substrate binding pocket than other laccases [17,18]. Guo et al. cloned and expressed a novel CotA laccase from *Bacillus licheniformis* that converts AFB1 to AFQ1 and epi-AFQ1 [19]. Interestingly, Loi et al. also found that peroxidase converted AFB1 to AFQ1 [20]. Furthermore, many studies have verified that the toxicity of AFQ1 is one order of magnitude lower than that of AFB1 [21,22]. Typically, bacterial laccase exhibits higher thermal and alkaline stability than fungus laccase and other laccase [23]. Therefore, bacterial laccase is a promising biocatalyst to degrade AFB1 in feed and food.

In this study, a strain of *Bacillus amyloliquefaciens* B10 with efficient AFB1-degrading activity was isolated from 74 strains, and the active substance responsible for AFB1 degradation by this strain was localised. A new laccase was found to efficiently degrade AFB1. The laccase was expressed in *E. coli* and the degradation characteristics of recombinant laccase were determined. This study provides a theoretical basis for AFB1 degradation by laccase and promotes the development of the enzymatic degradation of AFB1 in feed and food.

## 2. Results

### 2.1. Isolation and Identification of AFB1-Degrading Bacteria

**Figure 1:**
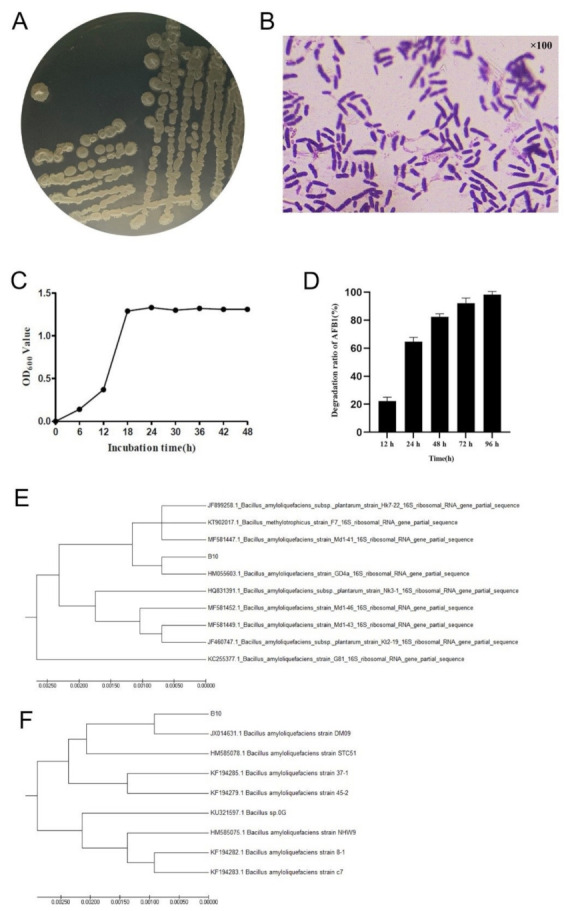
Characteristics of strain B10 and efficiency of AFB1 degradation: (**A**) Morphology of strain B10 cultured on LB medium for 24 h. (**B**) Gram stain of strain B10. (**C**) Growth curve of strain B10. (**D**) Effect of co-culture time on the degradation efficiency of AFB1 (2.5 μg/mL). (**E**) Phylogenetic tree of the 16S rDNA gene sequence of strain B10 using the neighbour-joining method. (**F**) Phylogenetic tree of the *gyrB* gene sequence of strain B10 using the neighbour-joining method.

The initial screening of the 74 strains kept in the laboratory revealed that the strain labelled B10 grew relatively well in the medium containing different concentrations of AFB1. Strain B10 was white on LB (Luria-Bertani) medium, slightly elevated, with a rough surface and folded colonies; single colonies were round and 3–4 mm in diameter (Figure 1A). Gram staining was positive with bluntly rounded ends, and rod-shaped cells were seen (Figure 1B). The complete 16S rDNA gene of length 1462 bp and the complete *gyrB* gene of size 1186 bp were obtained through amplification and sequencing with 16S rDNA and *gyrB* universal primers. Phylogenetic analysis of the 16S rDNA gene showed that the homology of B10 with the *Bacillus amyloliquefaciens* strain GD4a was 98.8%. Phylogenetic analysis of the *gyrB* gene implied that B10 was 99.8% homologous to the *Bacillus amyloliquefaciens* strain JX014631.1 on the same branch (Figure 1E,F). This strain was identified as *Bacillus amyloliquefaciens* based on the biochemical response characteristics (Table 1), combining morphological and phylogenetic characteristics. The OD_600_ value of strain B10 was also measured using a UV spectrophotometer at 6 h intervals. The results indicated that the strain entered the log phase at 12–18 h of incubation (Figure 1C). In further AFB1 degradation tests, the efficiency of AFB1 degradation increased significantly with the increase in co-culture time; almost complete degradation of 2.5 μg/mL AFB1 was found after 96 h of co-culture (Figure 1D).

### 2.2. Localisation of Degradation Active Substances

**Figure 2:**
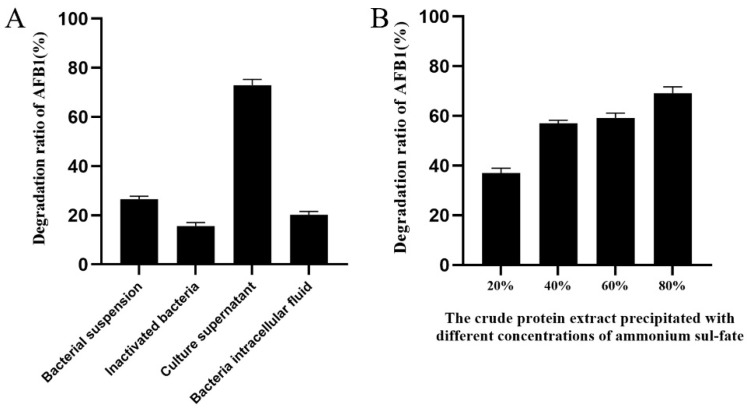
Localization of active substances for aflatoxin B1 degradation by strain B10: (**A**) Evaluation on the degradation effect of AFB1 (2.5 μg/mL) by each component of strain B10. (**B**) Evaluation on the degradation effect of AFB1 (2.5 μg/mL) by the crude protein extract of culture supernatant with different concentrations of ammonium sulfate.

The high-performance liquid chromatography (HPLC) results showed that the extracellular fluid of *B. amyloliquefaciens* B10 had the best degradation ability on AFB1, with a degradation rate of 72.9%. The other components of strain B10 caused a minor AFB1 degradation at a level far inferior to the extracellular fluid effect. Bacteria inactivated by high temperature could degrade 15.2% of AFB1, probably due to the physical adsorption of the cell membrane of the B10 strain (Figure 2A).

The crude protein of the supernatant from strain B10 was extracted with different concentrations of ammonium sulfate and dialysed for desalination. The degradation efficiency of AFB1 was 69.13% when the ammonium sulfate crude protein concentration was 80%. (Figure 2B). Therefore, we further determined that the AFB1-degrading substance might be a type of enzyme.

### 2.3. Isolation of AFB1-Degrading Proteins

**Figure 3:**
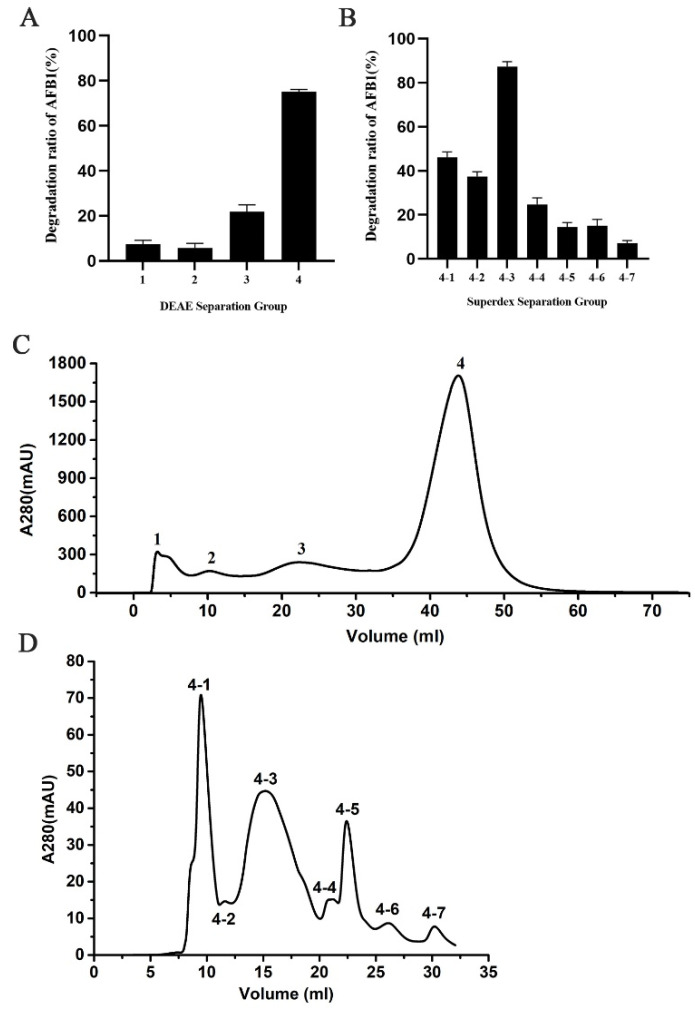
Isolation of AFB1 degrading enzyme from strain B10: (**A**) Degradation efficiency of AFB1 by each protein component precipitated by DEAE. (**B**) The efficiency of degradation of AFB1 by protein component precipitated by Superdex separation. (**C**) DEAE separation chromatogram (peaks 1 to 4 correspond to different protein fractions). (**D**) Superdex separation chromatogram (peaks 4-1 to 4-7 correspond to different protein fractions).

The crude protein from the extracellular fluid was concentrated and initially purified using a DEAE ion-exchange chromatography column to obtain an elution curve with four component peaks (Figure 3D). The degradation of AFB1 by each component showed that components 3 and 4 exhibited vigorous AFB1 degradation activity after 36 h incubation, and component 4 had the highest degradation activity with regard to AFB1 with a rate of 73.4% (Figure 3A). Therefore, component 4 was further purified by gel filtration chromatography and seven component peaks were obtained (Figure 3E). Among the seven protein components, components 4-3 demonstrated the highest degradation activity to AFB1 with a rate of 87.3% (Figure 3B). After SDS-PAGE and Coomassie brilliant blue R-250 staining of components 4-3, an obvious protein band of 35 kDa was observed. Components 4-3 were identified by protein mass spectrometry and matched to 60 proteins. The laccase component was contained in these 60 proteins by protein blast in NCBI. Therefore, we speculated that the proteins with AFB1 degradation activity in strain B10 probably contained laccase. However, the protein profile did not include a single protein, and whether all other proteins have AFB1-degrading activity warrants further research.

### 2.4. Molecular Docking for Function Prediction

**Figure 4:**
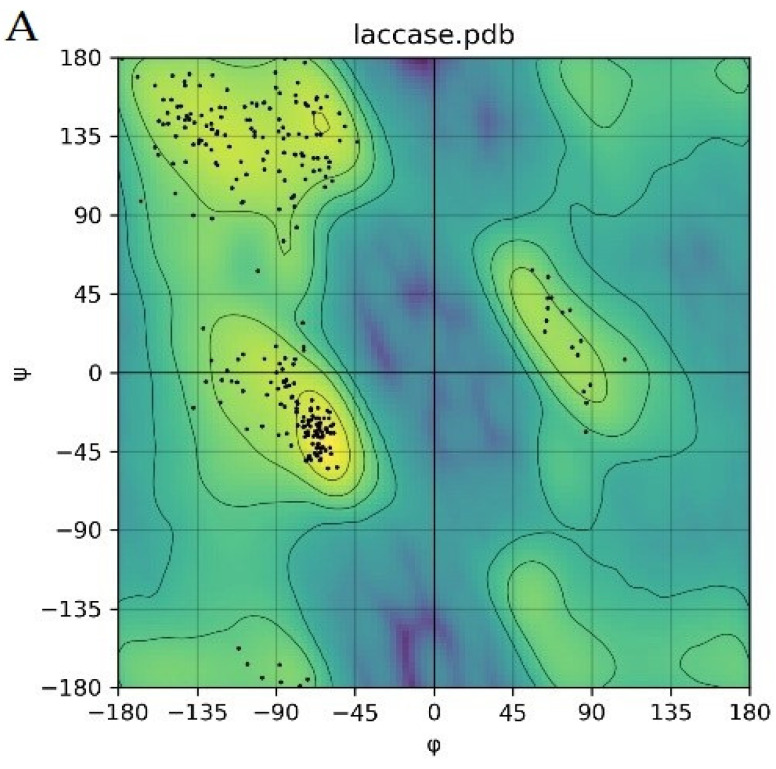

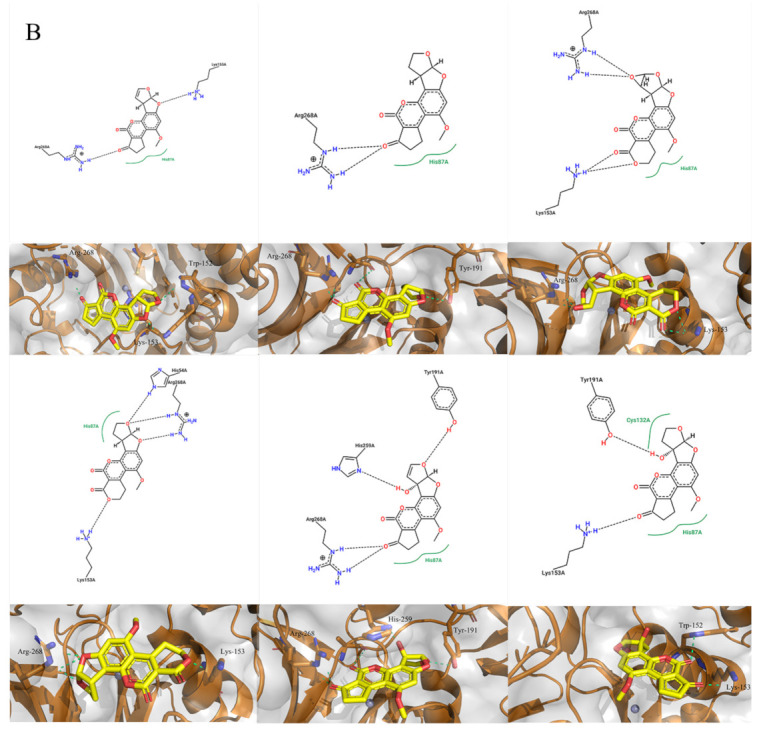
Molecular docking of aflatoxins with B10 laccase: (**A**) Ramachandran diagram of B10 laccase protein structure. (**B**) Models of the interaction of AFB1, AFB2, AFG1, AFG2, AFM1, and AFM2 with B10 laccase (grey, Zn^2+^; yellow, ligand).

Due to the excellent results achieved by AlphaFold2 in protein structure prediction [24], the predicted structure of a laccase from strain B10 was further refined and evaluated by Rasch plot, and 97% of the residues fell within the permissible interval (Figure 4A). This finding indicated that our predicted structure of B10 laccase could be used for molecular docking studies.

The molecular docking of the six aflatoxins (AFB1, AFB2, AFG1, AFG2, AFM1, and AFM2) was performed using the B10 laccase prediction model, and each aflatoxin had a high docking score with B10 laccase (Table 2, Figure 4B). Therefore, it is important to further verify the aflatoxin degradation ability of B10 laccase. The differences in docking scores with B10 laccase were also minor as the chemical structures of the six aflatoxins were similar. The docking score results indicated that AFB2 had the most vital binding capacity for laccase. Both Lys-153 and Arg-268 in B10 laccase produced hydrogen-bonding interactions with each aflatoxin, and these two residues were most likely to be the key amino acids for binding the toxins. His-87 made hydrophobic interactions with each aflatoxin, while His-87 was one of the amino acids with a coordination bond with Zn^2+^. The O-1 site on the terminal furan ring of AFB1 that acted as an acceptor with O-5 on the five-membered ring of AFB2 formed two hydrogen bonds as an acceptor with the side chain of Arg-268 and with Tyr-191. AFM1 formed hydrogen bonds with Arg-268, His-259, and Tyr-191. AFM2 formed hydrogen bonds with Trp-152, Lys-153, and Tyr-191 (Table 2, Figure 4B). The results of the molecular docking analysis showed that B10 laccase had a stable binding mode with aflatoxin, which was predicted to play a role in the degradation of aflatoxin. Therefore, we continued to clone the B10 laccase gene and investigated its protein expression in order to verify the ability of B10 laccase to degrade aflatoxin.

### 2.5. Cloning, Expression, and Purification of Laccase from Strain B10

**Figure 5:**
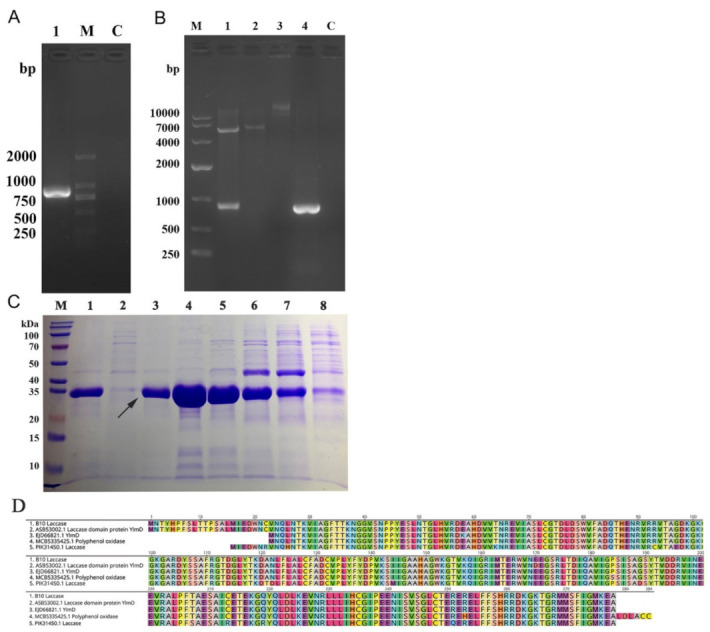
Gene cloning, expression, purification, and identification of B10 laccase: (**A**) Agarose gel electrophoresis to isolate the B10 laccase gene (M, marker; 1, B10 laccase PCR product; C, blank control). (**B**) Agarose gel electrophoresis to validate the results of pET-28a/B10 laccase recombinant vector (M, marker; 1, pET-28a/B10 laccase recombinant vector double digestion; 2, pET-28a double digestion; 3, pET-28a; 4, B10 laccase gene; C, blank control). (**C**) SDS-PAGE analysis of purified recombinant B10 laccase (M, marker; 1, beads; 2, 300 mM imidazole; 3, 200 mM imidazole; 4, 150 mM imidazole; 5, 100 mM imidazole; 6, 50 mM imidazole; 7, 10 mM imidazole; 8, imidazole-free buffer). (**D**) Amino acid sequence alignment of B10 laccase with *Bacillus amyloliquefaciens* 629 laccase, *Bacillus velezensis* RC218 laccase, and *Bacillus* 916 laccase.

The amino acid sequence of B10 laccase obtained in Section 2.3 was matched by NCBI BLAST and had the highest similarity with laccase No. ASB53002.1. The cloning primers were designed according to the base sequence of the CDs region of ASB53002.1 laccase, and the B10 laccase gene was cloned from strain B10 (Figure 4A). The open reading frame of the B10 laccase gene was 837 bp (Figure 5A), encoded 278 amino acids, and was predicted to have a molecular weight of 30.9 kDa and an isoelectric point 5.84. The amino acid homology of laccase cloned from the B10 strain to *Bacillus amyloliquefaciens* 629 laccase was 99.64% (GenBank: KNX34508.1) [25], to *Bacillus velezensis* RC218 laccase was 99.28% (GenBank: KUP42711.1) [26], and to *Bacillus* 916 laccase was 99.22% (GenBank: AIW29756.1) (Figure 5D) [27].

The laccase gene was transformed into *E. coli* DH5α and verified by double digestion (Figure 5B) and expressed in *E. coli* BL21(DE3). The purified recombinant laccase was then purified by Ni Sepharose 6 Fast Flow affinity chromatographic packing, and the purified recombinant laccase demonstrated a more distinct, but single, band by SDS-PAGE gel electrophoresis. The apparent molecular weight of the band was approximately 35 kDa, which was close to the predicted molecular weight (Figure 5C).

### 2.6. Efficiency of AFB1 Degradation by the Recombinant Laccase and the Effects of Different Conditions on AFB1 Degradation

**Figure 6:**
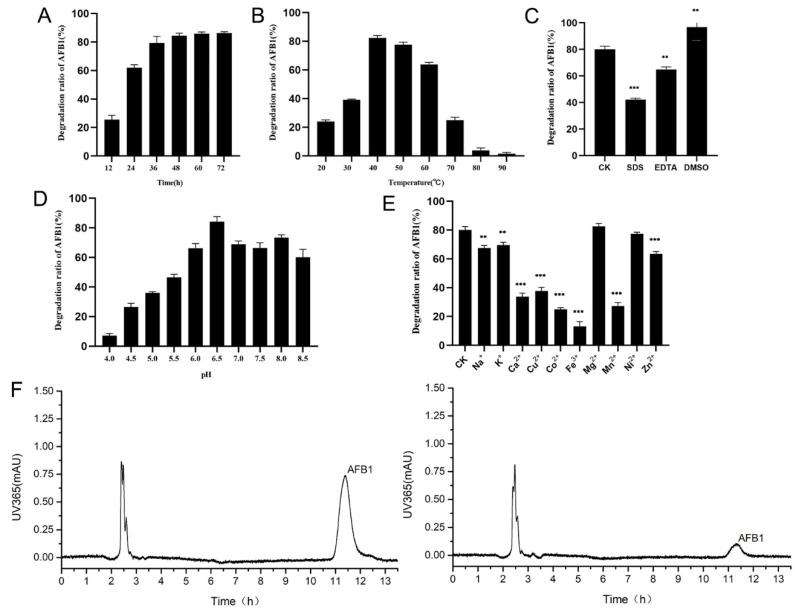
The efficiency of B10 laccase in degrading AFB1 and the effects of different conditions on the degradation: (**A**) Effect of co-culture time on the degradation of AFB1 by B10 laccase. (**B**) Effect of temperature on the degradation of AFB1 by B10 laccase. (**C**) Effect of common solutions on the degradation of AFB1 by B10 laccase (CK: In the control group, no substance was added). (**D**) Effect of pH on the degradation of AFB1 by B10 laccase. (**E**) Effect of metal ions on the degradation of AFB1 by B10 laccase (CK: In the control group, no metal ions was added). (**F**) Liquid chromatogram of AFB1 at 36 h co-culture (left, blank control; right, 36 h co-culture). **: *p* < 0.05; ***: *p* < 0.001.

A series of experiments proved that the recombinant laccase of strain B10 could efficiently degrade AFB1. The rate of degradation of AFB1 gradually increased with the increase in co-culture time, reaching 79.3% after 36 h. Meanwhile, the degradation rate of AFB1 reached a plateau at 48 h (Figure 6A,F). The results with regard to the effects of temperature on the degradation of AFB1 by the recombinant laccase showed that the degradation rate of AFB1 was gradually increased with the increase in temperature from 20 to 40 °C at pH 7.2; the degradation rate of AFB1 reached 82.4% at 40 °C. The degradation rate of AFB1 decreased rapidly because of the high temperatures ranging from 70 to 90 °C. However, the degradation rate of AFB1 exceeded 65% between 30 and 60 °C (Figure 6B). pH value had a strong influence on the degradation of AFB1. At pH 6.5, the degradation rate of AFB1 was the highest, reaching 84.2%. Under acidic conditions, the degradation rate of AFB1 was lower; at pH 4.0, the degradation rate was only 7.1%. From pH 6.0 to pH 8.0, the degradation rate of AFB1 exceeded 60% (Figure 6D). As AFB1 was efficiently degraded under strongly alkaline conditions, the effects of a pH greater than 9.0 on the degradation of AFB1 by the recombinant laccase were not considered in the present study.

The results with regard to the effects of different metal ions on the degradation of AFB1 by the recombinant laccase indicated that Ca^2+^, Cu^2+^, Co^2+^, Fe^3+^, Mn^2+^, and Zn^2+^ significantly reduced the degradation activity of AFB1 by the recombinant laccase compared with the control group with an 80.3% degradation rate of AFB1 (*p* < 0.001), while Na^+^ and K^+^ slightly reduced the degradation rate of AFB1 (*p* < 0.05). The metal ion Ni^2+^ had no significant effect on the degradation activity of AFB1 (Figure 6E). It was also shown that the metal chelators EDTA (*p* < 0.05) and SDS (*p* < 0.001) significantly reduced the AFB1-degrading activity of the recombinant laccase (Figure 6C), and the AFB1-degrading efficiency was significantly increased with the addition of DMSO (*p* < 0.05).

### 2.7. Site-Specific Mutagenesis of B10 Laccases

**Figure 7:**
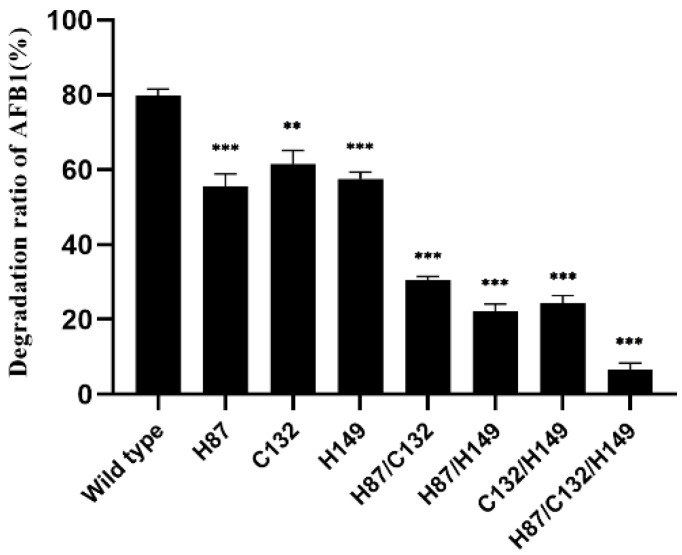
Effects on AFB1 degradation activity of B10 laccase mutants. **: *p* < 0.05; ***: *p* < 0.001.

The amino acid sequence of B10 laccase was matched to asb53002 by the NCBI protein database BLAST. The laccase domain protein YlmD score of 1 was 99.64%. After query, the annotation of the GenBank CDs region was directed to uniprotkb o31726. We found that B10 laccase contained three Zn^2+^ binding sites: namely, H-87, C-132, and H-149. Therefore, the roles of these three residues in laccase were assessed by site-specific mutagenesis. There were seven mutant proteins, single mutant H87, C132, and H149; double mutant H87/C132, H87/H149, and C132/H149; and triple mutant H87/C132/H149. These proteins were obtained by the polymerase chain reaction (PCR) method to mutate these sites to alanine. The results showed that after expression and purification, single mutants H87, C132, and H149 could be analysed by measuring AFB1-degrading activity. The AFB1-degrading activity of the single and double mutants H87, C132, H149, H87/C132, H87/H149, and C132/H149 was significantly decreased compared with the wild type, while the AFB1-degrading activity was further weakened by the triple mutant H87/C132/H149. In contrast, the triple mutant H87/C132/H149 decreased the degradation rate of AFB1 to 6.7% (Figure 7).

## 3. Discussion

Aflatoxin is of widespread concern due to its high toxicity, with approximately 4.5 billion people worldwide chronically exposed to aflatoxin through contaminated food [28,29]. In addition, data show that the consumption of aflatoxin-contaminated food is expected to further increase in incidence due to the ongoing COVID-19 pandemic, which has made the management of food and feed more difficult and complex [30]. The detoxification of aflatoxins by microorganisms is a promising new technology with broad application prospects. The control of aflatoxin by microorganisms mainly includes the inhibition of aflatoxin production, the adsorption of aflatoxin, and the degradation of aflatoxin [11]. To date, strains that are highly efficient in degrading aflatoxins have been isolated from various environmental sample species, including fungi, bacteria, actinomycetes, and protozoa [31]. In this study, 74 strains were screened in the laboratory, and one strain (laboratory number B10), was found to be highly efficient in degrading AFB1. The strain was identified morphologically, characterized biochemically, and analysed in a phylogenetic tree as *Bacillus amyloliquefaciens*. The B10 strain was 99.8% homologous to the *Bacillus amyloliquefaciens* strain JX014631.1 on the same branch and 98.8% homologous to the *Bacillus amyloliquefaciens* strain GD4a. Xu et al. isolated a strain of *Bacillus shark ii* L7 from 43 strains of bacteria, which could degrade 92.1% of AFB1 at a final concentration of 100 µg/L for 72 h at 37 °C. Further experiments implied that the active substance degrading AFB1 of strain L7 was mainly present in the culture supernatant, which could degrade 77.9% of the AFB1 within 72 h [32]. This was similar to the results of this study, where the supernatant of strain B10 could degrade AFB1 by 72.9% at 24 h. In contrast, the degradation rate of AFB1 was lower in the high-temperature inactivation group, the intracellular fluid group, and the bacterial suspension group compared with the culture supernatant. The high-temperature inactivation group presented the degradation of AFB1 probably due to the physical adsorption capacity of the cell membrane of the B10 strain. The presence of active substances in culture supernatants for AFB1 degradation by *Bacillus* was also revealed in tests by Gayatri [33], Wang [34], and Shu [35]. In this study, we found that protein precipitated by an ammonium sulfate gradient had the highest AFB1-degrading efficiency, so the AFB1-degrading substance was likely to be an enzyme or several enzymes in the culture supernatant.

In this study, the protein precipitated by ammonium sulfate was further purified, and the active component was measured through separation by DEAE anion exchange chromatography and gel filtration chromatography. A novel laccase that can efficiently degrade AFB1 was identified by protein mass spectrometry. The laccase gene had an open reading frame of 837 bp, an apparent molecular weight of 35 kDa, and a predicted isoelectric point of 5.84, and encoded 278 amino acids. The laccase cloned from strain B10 showed 99.64% amino acid homology to *Bacillus amyloliquefaciens* 629 laccase [25], 99.28% to *Bacillus velezensis* RC218 laccase [26], and 99.22% to *Bacillus* 916 laccase [27]. Proteins with AFB1-degrading activity were previously isolated using similar methods from culture supernatants of *Aspergillus flavus* ANSM068 [36], and the edible fungus *Pleurotus ostreatus* [37].

Superior results in all previous applications were achieved when Alphafold2 was used to predict the structure of the B10 laccase protein, and the release of Deepmind’s Alphafold2 software has ushered in a new revolution in high-quality three-dimensional (3D) protein structure prediction [38,39,40]. The predicted 3D model of B10 laccase was evaluated by structural refinement with a Rasch diagram, which allowed us to examine the model of the interaction of B10 and laccase with the substrate through molecular docking simulations without resolving the crystal structure. The active pocket of laccase appeared semi-open, with Zn^2+^ at the bottom of the pocket interacting with His-87, C-132, and H-149. This leads to speculation that Zn^2+^ may also be complexed with substrate or water molecules. In molecular docking, an implicit water model was used to approximate the solvent interaction, and a high degree of precision was employed to reveal the possible conformation of the ligand in the pocket. The docking results showed that both Lys-153 and Arg-268 could exert hydrogen bonding forces with aflatoxin, and therefore these two residues play an essential role in the binding of aflatoxin to B10 laccase. Notably, His-87 is known to play an essential role in catalysing the degradation of AFB1 by B10 laccase using a targeted mutation assay, and the docking results also indicated that His-87 can exert hydrophobic forces with aflatoxin, so His-87 is equally important in the binding of B10 laccase and substrate. The amino acid residues involved in hydrogen bonding were considered key residues for the interaction of laccase with specific ligands [41]. Lys-153 and Arg-268 will therefore be the focus of our future studies.

B10 laccase and aflatoxin have high docking scores. Therefore, we continue to verify the efficiency of B10 laccase in degrading aflatoxin. The efficiency of AFB1 degradation by B10 laccase was significantly different at different temperatures. The maximum degradation activity of AFB1 by this laccase reached 82.4% at 40 °C. Unlike the laccase identified by other researchers, the degradation activity of AFB1 decreased at temperatures ranging from 60 to 90 °C. The degradation rate of AFB1 by laccase isolated from *Stenotrophomonas* sp. CW117 was less than 60% at 40 °C but increased rapidly when treated at a high temperature for a short period; the degradation rate of AFB1 exceeded 84.6% at above 70 °C [31]. This may be due to evolutionary factors in the strain that led to the evolution of the laccase into a heat-resistant enzyme [42]. The highest AFB1-degradation activity of B10 laccase was achieved at pH 6.5, with a rate of degradation of 79.2%. The AFB1-degrading activity of this enzyme was deficient when pH was below 4.5, yielding a result more similar to that of Guo et al. [19]. The laccase isolated by Cai et al. exhibited low AFB1-degrading activity at pH 7–8 [31]. Since AFB1 was also efficiently degraded under strongly alkaline conditions, the effects of conditions above pH 9.0 on the degradation of AFB1 by recombinant laccase were not considered herein. Laccase activity could be increased at pH 9.8, but this condition was difficult to apply in food and feed detoxification [43]. In this study, we found that the addition of all metal ions exerted a negative effect on AFB1 degradation by B10 laccase, except for Mg^2+^, which enhanced the AFB1-degrading activity of B10 laccase. Notably, the addition of Cu^2+^ significantly reduced the AFB1-degradation activity of B10 laccase. This finding was consistent with results that laccase activity can be enhanced by 0.4–1 mM Cu^2+^ but was inhibited by high concentrations of Cu^2+^ [44]. In the present study, the organic solvent DMSO significantly improved the degradation activity of AFB1, in line with the findings of Wu al. [45], which may be attributed to changes in enzymatic activity due to the altered microenvironment caused by the organic solvent [46].

The residues H87, C132, and H149 of B10 laccase are the three metal-binding sites of this enzyme. The metal-binding sites of laccase are associated with the catalytic degradation of AFB1. When supplemented with metal ion chaperones during the expression of laccase, there was an increase in the metal-ion content of laccase and an improvement in its specific activity [47]. Previous research showed that the ribose portion of such proteins was coordinated by the side chains of these three residues. One of the conserved triplets of B10 laccase was predicted to be the active site, with similar results to the YlmD protein [48,49]. In the presence or absence of excess metal ions, the conformation of the protein was found to be significantly altered, including the R-loop, MR helix, and metal site centre, which destabilized the structure of the protein [50]. Although this does not change the overall structure, it can significantly affect enzymatic activity [51,52]. The present study showed that these three residues are essential for the degradation of AFB1 by B10 laccase. When these residues were replaced by alanine, the AFB1-degrading activity of the mutants was significantly decreased compared with the wild type.

## 4. Conclusions

In this study, a strain of *Bacillus amyloliquefaciens* B10 with high AFB1-degradation capacity was obtained and the active component of AFB1 degradation was located in the culture supernatant of this strain. A new laccase with high AFB1 degradation activity was obtained by the crude extraction of culture supernatant proteins with ammonium sulfate, DEAE, and gel filtration chromatography. The molecular docking results showed higher scores. Meanwhile, it was speculated that Lys-153, Arg-268, and His-87 residues in this laccase played an essential role in the binding of aflatoxin to this laccase. The B10 laccase gene was cloned from the bacterial genome and was expressed in *E. coli*. The recombinant laccase showed the highest AFB1-degradation activity at 40 °C and pH 6.5. The mutation of three key metal sites of the laccase implied that the AFB1-degradation activity of the triple mutants was almost wholly lost. These results suggested that B10 laccase is a promising enzyme for aflatoxin degradation. Our laboratory will continue to explore B10 laccase to improve enzymatic activity and stability, with a view to prompting the application of this enzyme in practical production as soon as possible.

## 5. Materials and Methods

### 5.1. Chemicals and Reagents

Gram stain, TAE, nucleic acid dye, Komas Brilliant Blue R-250, and ammonium sulfate were purchased from Beijing Solarbio Science & Technology Co., Ltd. (Beijing, China); the plasmid extraction kit, gel recovery kit, and PCR product recovery kit were purchased from Thermo Fisher Scientific (Waltham, MA, USA); DH5α and BL21 (DE3) receptor cells were sourced from TransGen Biotech (Beijing, China); point mutation kits from TransGen Biotech (Beijing, China) were used; point-mutation kits were purchased from Vazyme Biotech Co., Ltd. (Beijing, China); HiTrap Capto DEAE (5 mL), Superdex^TM^ 75 Increase 10/300 GL, and ÄKTA protein purification system were purchased from Cytiva (Washington, USA); and AFB1 was sourced from Sigma-Aldrich (Shanghai, China). All other reagents were at least of analytical purity.

### 5.2. Isolation of AFB1-Degrading Strains

Each of the 74 strains stored at −80 °C in the laboratory was inoculated into tubes containing 5 mL of LB liquid medium and incubated overnight at 37 °C on a shaker at 150 rpm. An inoculation loop was adopted to pick up a certain number of bacteria from the overnight culture of the strains to be tested on an LB agar gel plate for scribing. The strain was allowed to grow as a single colony for use, or the culture was purified if there were any stray colonies.

The purified strains to be tested were compared with the final concentration of 2.5 μg/mL AFB1 standard cultured in LB medium at 37 °C and 150 rpm for 24 h. The growth status of the strains in medium containing AFB1 was observed, and the strains that grew well were tested sequentially and at final concentrations of 5 and 10 μg/mL in AFB1 standard co-culture.

After initial screening, a laboratory strain labelled B10 grew well in AFB1 medium containing different concentrations of AFB1. All the components were incubated at 37 °C and 150 rpm for 12, 24, 48, and 96 h. The content of AFB1 was determined by high-performance liquid chromatography and ultraviolet (HPLC, UV) methods, and the degradation efficiency of strain B10 was calculated.

Chromatographic conditions: The chromatographic column was a C18 reverse adsorption column (4.6 mm × 250 mm, 5 μm), with an injection volume of 20 mL, a mobile phase of a 1:1 (*v*/*v*) water:methanol mixture, a flow rate of 1 mL/min, and a detection wavelength of 365 nm. The standard curve for the HPLC detection of AFB1 was *y* = 0.7735*x* + 0.1521, *R*^2^ = 0.9975.

### 5.3. Localisation of AFB1-Degrading Active Substances by Strain B10

The degradation of active substances from the fermentation broth of strain B10 was localised in four fractions: 500 mL of fermentation broth was taken and centrifuged through a low-temperature high-speed centrifuge at 4 °C, 8000 rpm for 10 min. The separate of the supernatant of the fermentation broth was separated from the bacterial precipitation, and 3 mL was taken as the cell secretion group (extracellular fluid group). The bacterial sediment was resuspended with 20 mL of sterile PBS buffer; we centrifuged and poured some PBS and washed it thrice before resuspension in 20 mL of sterile PBS. Afterwards, 3 mL was taken as the live-cell group (bacterial suspension group). Of the bacterial suspension, 3 mL was placed in an autoclave at 121 °C for 30 min and allowed to cool before use (inactivation group). The remaining bacterial suspension was dosed with PMSF at a final concentration of 1%, crushed in a low-temperature ultrasonic cell crusher, then centrifuged at 4 °C, 13,000 rpm for 30 min. Each component was incubated with AFB1 standard at a final concentration of 2.5 μg/mL for 24 h in an incubator at 37 °C, and the degradation rate of each component against AFB1 was measured using HPLC.

### 5.4. Crude Extraction of Protein from Culture Medium

The B10 strain was inoculated in four vials of LB medium and incubated overnight at 37 °C. The supernatant was removed by high-speed low-temperature centrifugation, and ammonium sulfate was added to give final concentrations of 20, 40, 60, and 80%. After thorough mixing with a magnetic stirrer, the culture was kept at 4 °C overnight. The supernatant of each group was centrifuged at 13,000 rpm for 30 min at 4 °C. The supernatant was discarded, and the residue was dissolved in 10 mL of sterile PBS, transferred to a dialysis bag with a cut-off volume of 3.5 kDa, and dialysed in PBS buffer for 24 h at 4 °C.

One millilitre of crude protein solution of each component after dialysis was taken separately, filtered through a 0.22 μL needle filter, and incubated with 500 μL of AFB1 standard at a final concentration of 2.5 μg/mL for 24 h. The rate of degradation of each component was measured using HPLC.

### 5.5. Isolation and Identification of AFB1-Degrading Proteins

The crude extracted protein from the fermentation broth culture supernatant was concentrated and initially purified by passing through a DEAE ion-exchange column. The crude proteins were loaded onto a column pre-equilibrated with buffer (containing 20 mM/L Tris, 10 mM/L NaCl, pH 8.0) and eluted with a 0.01–2.0 M/L linear concentration gradient NaCl buffer at a flow rate of 1 mL/min. The eluate was collected separately according to the absorption peaks. Each component was incubated with 500 μL of AFB1 standard at a final concentration of 2.5 μg/mL for 24 h. The efficiency of each component in degrading AFB1 was obtained using HPLC.

The fraction with good degradation was purified by gel filtration chromatography. The solution to be measured was loaded onto a column pre-equilibrated with buffer (containing 20 mM/L Tris, 50 mM/L NaCl, pH 8.0) and eluted at a flow rate of 0.2 mL/min. The eluate was collected separately according to the absorption peaks. The degradation rate of each group was detected as described. The components with the best degradation effect were analysed by SDS-PAGE, and the proteins in the optimal components were identified by protein mass spectrometry.

### 5.6. Structural Modelling of the Recombinant Laccase and Molecular Docking

Through recombinant laccase amino acid sequencing using AlphaFold2 on the Beijing Supercomputing Cloud N22 partition with a full_dbs preset [25], a total of five initial models and five models relaxed by the Amber relaxation procedure were generated, after which the highest-ranked conformation was selected for subsequent docking analysis based on the average plDDT ranking. The recombinant laccase’s optimal structure was plotted in Python 3.8 using the RamachanDraw package for quality and poor contact assessment between residues.

As retrieved from AFB1 (PubChem CID: 186907), AFB2 (PubChem CID: 2724360), AFG1 (PubChem CID:133065469), AFG2 (PubChem CID: 2724362), AFM1 (PubChem CID: 15558498), and AFM2 (PubChem CID: 23318), six molecules were downloaded and saved in SDF format, semi-empirically optimized using MOPAC2016 (http://openmopac.net/, accessed on 15 November 2020) under the PM7 PRNT = 2 parameter. Optimized structures were prepared using the MGLTools 1.57 suite of prepare_ligand.py which could be converted to pdbqt format to give the Gasteiger charge and retain the total hydrogen.

The prediction of the recombinant laccase Zn^2+^ binding site using the bioinformatics tool UniProt (EMBL-EBI, Cambridge, UK) was followed by use of the NCBI BLAST tool (National Center for Biotechnology Information, Bethesda, MD, USA). The PDB database was selected, the crystal structure of a purine nucleoside phosphorylase with 49.40% homology (PDB ID: 6T0Y) was retrieved, the two structures were fitted in pymol2.5, the template Zn^2+^ was retained, the amino acids in the Zn^2+^ binding site were adjusted, and the MGLTools 1.57 suite was used to create a recombinant laccase pdbqt file containing charge and H atoms.

Docking studies of the recombinant laccase were performed using watvina (https://github.com/biocheming/watvina, accessed on 30 November 2021, Ximing Xu, Qingdao, China), a deeply optimized offshoot of vina, using the getbox plugin in pymol to generate docking boxes centred on the metal-binding site of the recombinant laccase. The docking box was centred at center_x = 0.8, center_y = −3.1, center_z = −5.7; size_x = 19.5, size_y = 21.2, size_z = 18.5, after which the molecule and recombinant laccase were docked using watvina, and the solvent interaction was approximated by using an implicit water model. The docking results were plotted using pymol.

### 5.7. Gene Cloning, Protein Expression, and Purification of the Recombinant Laccase

Strain B10 was cultured overnight in LB medium, and DNA was extracted from the strain using a DNA extraction kit. Amino acid sequence results obtained from protein mass spectrometry identification in 5.5 were aligned by NCBI BLAST. Cloning primers were designed based on the CDs region base sequences of the most similar proteins. The restriction enzymes BamH I and Xho I were selected, and the expression vector pET-28a was designed with fragment amplification primers as follows:

Lac-F: 5′CGGGATCCATGAATACATATCACCCGTTTAGTCTT3′

Lac-R: 5′CCCTCGAGTTATGCCTCCTTCATTCCGATAAAG3′.

PCR reaction conditions consisted of denaturation at 98 °C for 10 s, annealing at 55 °C for 5 s, extension at 72 °C for 30 s, 34 cycles from denaturation to extension steps, and storage at 4 °C. PCR fragments were recovered using an agarose gel DNA extraction kit. The double-cleaved B10 laccase gene fragment was inserted into the expression vector pET-28a, transformed into Trans5α chemically competent cells, coated onto LB medium containing 100 mg/mL kanamycin, and incubated overnight. Three positive clones were selected for sequencing, and the plasmids from the positive clones corresponding exactly to the B10 laccase gene were extracted and transformed into BL21 (DE3) chemically competent cells. Positive clones coated on LB medium containing 100 mg/mL kanamycin were inoculated in LB liquid medium. Expressed cells were collected by centrifugation and resuspended in sterile PBS, fragmented by ultrasonic cell disruptor, and purified by Ni Sepharose 6 FF affinity chromatography packing with His-tagged laccase in the supernatant. The purity of the recombinant laccase was determined by sodium dodecyl sulfate–polyacrylamide gel electrophoresis (SDS-PAGE), and the protein concentration was detected by bicinchoninic acid (BCA) assay. BCA standard curve regression equation was *y* = 0.7618*x* + 0.1371.

### 5.8. Efficiency of AFB1 Degradation by a Recombinant Laccase and Its Effect on AFB1-Degrading Activity under Different Conditions

A recombinant laccase (20 μg/mL) was incubated with AFB1 standard at a final concentration of 2.5 μg/mL for 12, 24, 36, 48, 60, and 72 h at pH 7.2 and 37 °C. The samples were centrifuged in 1.5 mL centrifuge tubes at 13,000 rpm for 5 min at room temperature, and the supernatant was removed from the tubes. A total of 500 μL of the supernatant was transferred to a new 1.5 mL sterile centrifuge tube, and all mixed samples were withdrawn using a 1 mL syringe and filtered through a 0.22 μm organic phase needle filter. A total of 20 μL of the filtrate was injected into the HPLC detection system using a microsampling needle (the same treatment was applied to the control). The samples were assayed for AFB1 according to the AFB1 standard curve.

To evaluate the effects of different temperatures on the degradation of AFB1 by the recombinant laccase from strain B10, the recombinant laccase (20 μg/mL) and AFB1 standard at a final concentration of 2.5 μg/mL were dissolved in PBS buffer at pH 7.2 and incubated at 20, 30, 40, 50, 60, 70, and 80 °C for a total of 36 h.

To characterize the effects of different pH values on the degradation of AFB1 by recombinant laccase of strain B10, the recombinant laccase (20 μg/mL) and the AFB1 standard at a final concentration of 2.5 μg/mL were incubated at pH 4, 5, 6, 7, 8, 9, and 10 for 36 h at 37 °C.

To determine the effects of different metal ions on the degradation of AFB1 by the recombinant laccase from strain B10, the recombinant laccase (20 μg/mL) and 2.5 μg/mL of AFB1 standard were dissolved in PBS buffer at pH 7.2 and incubated for 36 h at 37 °C with 10 mM Na^+^, K^+^, Co^2+^, Fe^3+^, Mg^2+^, Cu^2+^, Ca^2+^, Mn^2+^, Zn^2+^, and Ni^2+^ for 36 h.

To estimate the effects of other conditions on the degradation of AFB1 by the recombinant laccase of strain B10, the recombinant laccase (20 μg/mL) and the AFB1 standard at a final concentration of 2.5 μg/mL were dissolved in PBS buffer at pH 7.2 and incubated for 36 h at 37 °C in 10 mM ethylenediamine tetraacetic acid (EDTA), sodium dodecyl sulfate (SDS), and dimethyl sulfoxide (DMSO), respectively.

The PBS buffer was incubated with AFB1 standard at a final concentration of 2.5 μg/mL for the same length of time as the control group. HPLC was used to assay all such samples, and the content of AFB1 was determined according to the AFB1 standard curve.

### 5.9. Targeted Mutagenesis of the Recombinant Laccase

The amino acid sequence of the recombinant laccase was blasted through the UniProt database and the PDB database to determine proteins with a high matching score for this laccase, and its metalloid sites were recorded as H87, C132, and H149. Targeted mutations were achieved by the Fast Mutagenesis kit according to the instructions supplied with the kit. Mutation primers were as follows:

H87-F: 5′CCAGACAgctGAAAACCGCGTCCGGCGCGTGA3′

H87-R: 5′GGTTTTCagcTGTCTGGTCGGCGAACACCCAG3′

C132-F: 5′TGTTTTGCGGACgctGTGCCCTTGTATTTTTATGACCCG3′

C132-R: 5′ACagcGTCCGCAAAACAAAGGGCCAAAAAAAG3′

H149-F: 5′TTATCGGCGCTGCCgctGCCGGATGGAAGGGGACG3′

H149-R: 5′agcGGCAGCGCCGATAATGGATTTCACCGGGT3′.

The mutants were transformed into DH5α, and assays determined the correct sequence of the recombinant laccase mutant. The plasmid carrying the mutant was also transformed into *E. coli* BL21 receptor cells, and the purified mutant was expressed as described above, while its AFB1-degrading activity was determined.

## Figures and Tables

**Table 1:** Physiological and biochemical characteristics of strain B10.

Indicator	Result
Methyl Red assay	+
Anaerobic	−
Oxidase	+
Contact enzyme assay	+
V-P test	+
Indole test	−
Starch hydrolysis	+
Glucose fermentation	+
Glucose gas production	−
Gelatine liquefaction	+
Citrate	+
Xylose	+
Nitrate	+
Propionate	−
7% NaCl growth	+

V-P Test: Voges–Proskauer test.

**Table 2:** Molecular docking results.

Compound Name	Structure	Docking Score (kcal/mol)	Hydrogen Bond			Hydrophobic Effect
			Enzyme Residues	Distance D-A (Å)	Acceptor Atom	
AFB1	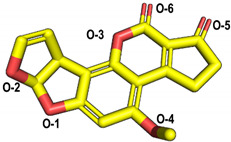	−5.60	Trp-152	3.17	O-2	His-87
			Lys-153	2.82	O-1	
			Arg-268	2.89	O-5	
AFB2	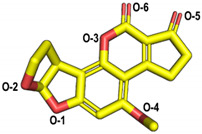	−6.82	Tyr-191	3.50	O-2	His-87
			Arg-268	2.63	O-5	
				2.62	O-5	
AFG1	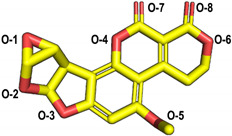	−6.58	Lys-153	2.84	O-6	His-87
				3.15	O-8	
			Arg-268	2.79	O-1	
				2.77	O-1	
AFG2	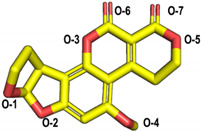	−5.31	Lys-153	2.77	O-5	His-87
			Arg-268	2.65	O-2	
				3.09	O-1	
AFM1	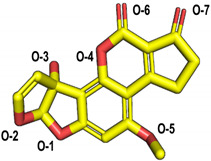	−6.30	Tyr-191	3.16	O-2	His-87
			His-259	2.87	O-3	
			Arg-268	2.84	O-7	
				2.82	O-7	
AFM2	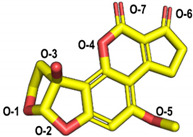	−6.13	Trp-152	2.78	O-7	His-87 Cys-132
			Lys-153	2.76	O-6	
			Tyr-191	2.65	O-3	

## Data Availability

The data presented in this study are available in this article.
